# Social Inequalities in Exposure to Air Pollution in the EPIC Cohorts of Turin and Varese

**DOI:** 10.3390/toxics13090724

**Published:** 2025-08-28

**Authors:** Mattia Costantino, Francesco Sera, Carlotta Sacerdote, Sabina Sieri, Valeria Pala, Fulvio Ricceri, Chiara Di Girolamo

**Affiliations:** 1 Dipartimento di Scienze Cliniche e Biologiche, Università degli Studi di Torino, Regione Gonzole 10, 10043 Orbassano, Italy; mattia.costantino@edu.unito.it (M.C.); chiara.digirolamo@unito.it (C.D.G.); 2 Department of Statistics, Computer Science and Applications ‘G. Parenti’, University of Florence, 50134 Firenze, Italy; francesco.sera@unifi.it; 3 Dipartimento di Scienze della Salute, Università degli Studi del Piemonte Orientale, Via Solaroli 17, 28100 Novara, Italy; 4 Epidemiology and Prevention Unit, Fondazione IRCCS Istituto Nazionale dei Tumori di Milano, Via Venezian, 1, 20133 Milan, Italy

**Keywords:** air pollution, health inequalities, EPIC, socioeconomic position, Italy

## Abstract

In Europe, evidence on the relationship between socioeconomic position (SEP) and air pollution exposure is mixed. We assessed the association between individual SEP (education and occupation) and air pollution in the Turin and Varese European Prospective Investigation into Cancer and Nutrition cohorts. This cross-sectional study included participants enrolled between 1992–1998, categorised by three educational (high, medium, and low) and three occupational (high-, medium-, and low-skilled) levels. Air pollution exposure (2008–2011) at residential addresses was estimated using Land Use Regression models. Nitrogen dioxide (NO_2_) and nitrogen oxide (NOx) data were available for both cohorts; particulate matter (PM_2.5_, PM_10_) only for Turin. Linear regression models (adjusted for sex, age, and marital status) estimated associations between SEP and annual mean pollutant concentrations (µg/m^3^), stratified by cohort. In Varese, lower education was associated with lower NOx exposure. In Turin, medium and low education were also linked to lower NOx exposure, though without a clear gradient. In both cohorts, individuals in medium- and low-skilled occupations had lower nitrogen exposure than those in high-skilled jobs. Associations between SEP and PM exposure in Turin were weak to null. In conclusion, lower SEP was associated with slightly lower nitrogen exposure; no clear link was found with PM.

## 1. Introduction

Clean air is essential for healthy living; however, in most parts of the world air is contaminated with many pollutants that adversely affect human health [1]. While initial attempts to tackle the issue began with the industrialisation process [2], it was not until the World Health Organization (WHO) published its first air pollution guidelines in 1987 [3], followed by numerous updates, that the problem gained significant attention. Consequently, Europe established air quality standards according to the 2005 WHO guidelines [4]. However, they were updated in 2021 with more ambitious goals, followed by a European Commission proposal to revise the Ambient Air Quality Directive [5]. Indeed, the impact of air pollution on public health is substantial, as highlighted in European Environmental Agency (EEA) reports [6]. According to their data, in 2021, 97% of urban residents in the EU-27 were exposed to air pollution levels exceeding the recommendation limits [7], resulting in 253,000 and 52,000 premature deaths attributable to PM_2.5_ and NO_2_ concentrations, respectively, surpassing WHO guidelines in 2021 [8].

Given the importance of this issue, it is crucial to investigate whether the pollutants’ distribution is uniform in the population or if distinct levels of exposure characterise different socioeconomic groups. Notably, the unequal distribution of most risk factors in the population [9], contributing to so-called health inequalities [10], underscores the importance of determining if this trend extends to the present case. In particular, contrary to North America, a unique pattern does not characterise European cities, each marked by its unique pollution exposure [11], and the role of different socioeconomic indicators remains to be determined, with European data demonstrating that the power of the association reduces when passing from national to ecological to individual indicators of socioeconomic deprivation [12].

In this context, this study aims to assess the relationship between two individual indicators of socioeconomic position (SEP) (education and occupational levels) and a selection of air pollutants (NOx, NO_2_, PM_10_, and PM_2.5_) in the Turin and Varese European Prospective Investigation into Cancer and Nutrition sub-cohorts.

## 2. Materials and Methods

### 2.1. The Study Design, Data Sources, and Study Population

The data came from the European Prospective Investigation on Cancer and Nutrition (EPIC), a multicentre European study in which more than half a million volunteers of both sexes and aged between 35 and 74 years at recruitment were recruited in 10 European countries between 1993 and 1998 [13,14]. For the present study, the population was restricted to a sub-sample of the Turin EPIC cohort (five municipalities within the metropolitan area of Turin: Collegno, Moncalieri, Grugliasco, Nichelino, and Rivoli, and the municipality of Turin) and the whole Varese EPIC cohort (entire province)—both located in Italy—because air pollution data were only available for these sub-populations. The two cohorts were recruited during the years 1993–1998 [15]. Each participant filled in a questionnaire on dietary information, reproductive history, physical activity, smoking and alcohol drinking history, medical history, and other socioeconomic variables (including occupation and educational attainment). Anthropometric measurements were also collected together with a blog sample stored in liquid nitrogen.

Air pollution data came from the European Study Cohorts for Air Pollution Effects (ESCAPE), an investigation into the long-term effects of exposure to air pollution on human health in Europe, which included 36 European areas (among others, a sub-sample of the EPIC Turin and the whole EPIC Varese) in which air pollution was measured and Land Use Regression models were developed to assign the annual mean concentration at the address of cohort members (declared at enrolment and transformed into geographical coordinates) [16].

### 2.2. Variables of Interest

#### 2.2.1. Outcomes

The outcome of interest is the level of a selection of pollutants, whose measurement unit is µg/m^3^. For both cohorts, the following pollutants were considered:

(1) Nitrogen oxides (NOx);
(2) Nitrogen dioxide (NO_2_).

Two additional pollutants were considered for the Turin cohort only:

(3) Particulate matter with an aerodynamic diameter of less than 2.5 μm (PM_2.5_);
(4) Particulate matter with an aerodynamic diameter of less than 10 μm (PM_10_).

All considered pollutants have been classified by the International Agency Against Cancer (IARC) in Group 1 (carcinogenic to humans) [17]. These specific pollutants were selected as part of the ESCAPE project protocol, which aimed to provide standardised and harmonised exposure data across multiple European cohorts. The selection was guided by both scientific relevance and practical feasibility: these pollutants are well-established indicators of traffic-related air pollution, exhibit strong spatial variability within urban environments, and have consistently shown associations with adverse health outcomes in epidemiological research [18]. Moreover, NO_2_, NOx, PM_2.5_, and PM_10_ were the only pollutants for which high-quality, spatially resolved exposure estimates were available across the study areas, enabling reliable comparisons between cohorts and cities. For each pollutant and for the two cohorts, the annual mean concentration in the study period at the address of each study participant was obtained from the ESCAPE database. According to the ESCAPE protocol, 40 measurement sites were selected for nitrogen molecules and 20 measurement sites for atmospheric particulate matter. At the selected sites, between October 2008 and February 2010 for the first group and between November 2009 and April 2011 for the second group (sites were stochastically divided into two groups for reasons of feasibility), three measurement periods of two weeks were used [19,20]. The periods were one during winter, one during summer, and one during an intermediate-temperature season (spring or autumn). In addition, sampling was performed in weeks with no unusual events (e.g., wildfires or school holidays). All samples were centrally analysed at a unique laboratory (IRAS at Utrecht University, Utrecht, The Netherlands). To account for the strong temporal variability of pollutant concentrations, measurements were adjusted using data from continuous monitoring stations located in the study area. Specifically, the difference between each two-week measurement and the annual mean concentration at a reference site was calculated and subtracted from the raw value, following established methods previously validated in the Traffic-related Air Pollution and Childhood Asthma (TRAPCA) study [21,22]. Moreover, Land Use Regression (LUR) models were developed for each pollutant in each study area using the adjusted yearly mean concentration as the dependent variable and geographical characteristics (e.g., population density, traffic intensity, industry, and proximity to harbours, etc.) as predictors [23,24]. These models have been used to estimate NOx, NO_2_, PM_10_, and PM_2.5_ at the address of each cohort member at enrolment, after appropriate transformation into geographical coordinates.

#### 2.2.2. Exposure and Confounding Variables

The primary exposure variable is the level of education, which has been used as the main indicator of individual SEP because it is considered to be a valid proxy for social position since it reflects life experiences during childhood and work opportunities in adulthood [25].

The variable was categorised into three groups:

- Low-level education (those who achieved at most a lower secondary school certificate);
- Medium-level education (at most a higher secondary school certificate);
- High-level education (more than a higher secondary school certificate).

The educational level variable was then transformed into the relative index of inequality (RII) [26], a metric previously computed for the EPIC cohorts [27]. This measure gauges inequality in a relative manner, designed to mitigate distortions arising from varying distributions of educational attainment across countries, sex, and age cohorts.

The RII was derived by associating each participant’s education level with the midpoint of the cumulative proportion of individuals sharing that same educational attainment within a specific category (sex, age, and cohort). This procedure was iterated for every education level within each distinct category. The resultant value assigned to each participant ranged from 0 (indicating minimal disadvantage, i.e., high education) to 1 (denoting substantial disadvantage, i.e., low education), effectively characterising their social standing relative to their counterparts within the designated category (sex, age, or cohort).

These computed values were subsequently utilised to harmonise data from diverse categories. Numerically ordering these values offers insight into the relative social positioning of each participant, disentangled from the influences of age, sex, and cohort [27].

For the purpose of this work, the overall distribution was then grouped in tertiles, and an RII score (high, middle, or low) was assigned to each participant based on the tertile comprising their RII value. This assignment corresponds to their position within the distribution: high for the first tertile, middle for the second tertile, and low for the third tertile. Importantly, this process can lead to individuals with differing education levels being grouped into the same RII tertile, accounting for the effect of sex, age, and cohort differences in terms of educational attainment.

The secondary exposure variable is the occupational level. As an individual indicator of SEP, the occupational level not only mirrors the education achieved to obtain that job and the income it provides, but also has its own role in determining the social status defined by one’s occupation [28]. It has been categorised into three groups according to the International Standard Classification of Occupations (ISCO) of the International Labour Organization (ILO) [29], as proposed in the LIFEPATH project [30]:

- Low-skilled employment (skill level 1): unskilled and skilled workers (ESCO categories 7, 8, and 9);
- Medium-skilled employment (skill level 2): farmers, retailers, and clerical workers (ESCO categories 4, 5, and 6);
- High-skilled employment (skill levels 3–4): professionals and managers (ESCO categories 1, 2, and 3).

For study participants still of a working age, the job at the time of enrolment was used, while for the retired ones, the last job before retirement was considered.

Unemployed people and housewives were excluded from the analysis.

Adjustment variables included sex (male or female), marital status (married or not married), and age classified into 10-year classes (35–44, 45–54, 55–64, and ≥65).

### 2.3. Statistical Analysis

#### 2.3.1. Descriptive Analyses

For continuous variables (pollutants), the mean, standard deviation, and median were computed. For categorical variables (RII tertiles, occupational level, age, sex, and marital status), absolute and relative frequencies were calculated. *T*-tests and ANOVA tests were conducted to test differences in continuous variables, and chi-square tests to evaluate associations among categorical variables.

#### 2.3.2. Models

After verifying the distribution’s normality with the skewness and kurtosis tests for normality [31,32,33], considering that the study involved more than 2000 subjects and after a visual data distribution inspection, it was decided to use linear regression models.

First, univariable linear regression models were run to evaluate the association between each pollutant and the main exposures (education and occupation level) and the confounding variables (age, sex, and marital status).

Secondly, for each pollutant, multivariable linear models adjusted for age, sex, and marital status were fitted for each exposure variable (education and occupation level).

The modifying effects of the cohort were tested in the fully adjusted models using a Wald test of hypotheses; given the presence of interaction, results were reported for the two cohorts separately.

Analyses were performed using Stata version 18, developed by StataCorp LLC (College Station, TX, USA) and released in 2023.

## 3. Results

The cohort comprised 19,842 subjects, 55.9% from the Varese cohort and 44.1% from the Turin cohort. The mean age of the pooled cohort was 50.5 years (51.1 years for Varese and 49.8 years for Turin). Males represented 36.6% of the pooled cohort, with substantial variation between the two cohorts: they were 21.4% of the total in the Varese cohort and the 55.8% of the total in the Turin one. The distribution of the RII tertiles of educational level was similar in the two cohorts, with the lower level of education always being the most common level (38.4% and 34.7% for Varese and Turin, respectively). The distribution of occupation differed between the two cohorts: more than half of the subjects in the Varese cohort (51.5%) had low-skilled employment, whereas more than half of the Turin one (59.0%) had medium-skilled employment. On the contrary, marital status had an almost identical distribution, with 86.8% and 86.1% of people married in Varese and Turin, respectively (Table 1).

The mean levels of nitrogen molecules (NO_2_ and NOx) were consistently higher in the Turin cohort (*p* < 0.01), with differences exceeding 20% for NO_2_ (43.18 µg/m^3^ for Varese and 54.16 µg/m^3^ for Turin) and 15% for NOx (85.38 for Varese and 99.55 µg/m^3^ for Turin). The mean concentrations of particulate matter, which were available only for the Turin cohort, were 46.51 µg/m^3^ and 30.14 µg/m^3^ for PM_10_ and PM_2.5_, respectively (Table 2).

There was a clear relationship between educational level and occupation (*p* < 0.001), with people with higher education levels having more skilled jobs than those with lower education levels (68.5% and 11.7% high-skilled jobs for higher and lower RII tertiles, respectively, when considering the whole cohort) (Table A1 in Appendix A). In both cohorts, women were generally less educated than men; however, in the Varese cohort, the difference between low-educated men and women was greater than in the Turin cohort (for males and females, respectively, 31.6% and 40.3% in Varese and 34.0% and 35.7% in Turin, with *p*-values < 0.01 in both cases). In general, there was strong evidence that the educational level was not evenly distributed across age groups (*p*-values were <0.01 both in Turin and in Varese). In both cohorts, older people were more likely to have a low education, and young age groups to have a medium or high education. The proportion of not married people was higher among those with a high level of education both in Varese (41.5%) and in Turin (36.4%) (*p* < 0.01).

Similarly to what was described for the educational level, in both cohorts, older age groups were more likely to be employed in low-skilled sectors, and young age groups in medium-skilled jobs (*p*-values were <0.01 both in Turin and in Varese) (Table A2 in Appendix A). On average, men had more high-skilled jobs than women (for males and females, respectively, 12.0% and 3.6% in Varese and 7.8% and 4.8% in Turin, with *p*-values < 0.01 in both cases). The association between occupational levels and marital status was weak in the Varese cohort (51.1% and 50.4% of low-skilled jobs in Varese for married and unmarried, respectively, with a *p*-value of 0.01) and stronger in the Turin one (34.0% and 26.8% of low-skilled jobs in Turin for married and unmarried, respectively, with a *p*-value < 0.01).

Considering the two nitrogen molecules, the association between the exposures and the outcomes has similar patterns within each cohort, with the more deprived exposed to slightly lower levels of air pollution (Table 3).

**Figure 1:**
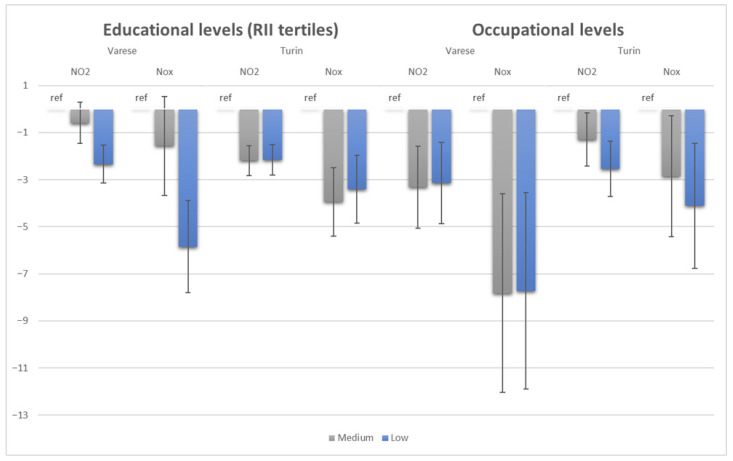
Beta coefficients (with 95% confidence intervals) of multivariate linear regression analysis with nitrogen molecules as outcomes and educational and occupational levels as exposures in Varese and Turin. The reference groups (ref) are the highly educated and the high-skilled occupational levels.

In the Varese cohort, the univariate analysis showed that the mean exposure to NO_2_ and NOx was significantly lower among the low-educated than among the highly educated (−1.67 CI 95% [−2.44; −0.90] for NO_2_ and −4.23, CI 95% [−6.09; −2.37] for NOx). The association became even stronger when the model was fully adjusted for the confounding factors (−2.34, CI 95% [−3.14; −1.53] for NO_2_ and −5.85, CI 95% [−7.80; −3.89] for NOx) (Figure 1). Looking at the effect of occupation, the univariate analysis showed that the mean exposure among people in both medium and low occupational levels was significantly lower than among those in high-skilled occupations, with a higher effect among medium occupational levels (−4.42, CI 95% [−6.03; −2.81] for NO_2_ and −10.33, CI 95% [−14.23; −6.44] for NOx for medium occupational levels). In the fully adjusted models, the point estimate did not change for the low occupational level, but shrank for the medium occupational level (−3.32, CI 95% [−5.06; −1.57] for NO_2_ and −7.82, CI 95% [−12.04; −3.60] for NOx for medium occupational levels).

In the Turin cohort, the univariate and multivariate analyses showed that the mean NO_2_ exposure among people with both medium and low educational levels was lower than the one among the highly educated, and that little confounding effect was present (in the multivariate analysis, −2.19, CI 95% [−2.84; −1.55] and −2.16, CI 95% [−2.80; −1.52] for medium and low levels of education, respectively). A similar pattern was observed for NOx. Besides highlighting the confounding effect of age (mainly among the older), these results reveal that the mean exposure to nitrogen molecules was significantly lower among the medium- and low-educated than among the high-educated counterpart, but without an evident gradient. Regarding the effect of occupation, a gradient according to which a decrease in occupational level corresponds to a decrease in exposure to NO_2_ and NOx was present in both univariable and multivariable analyses, with little confounding effect and with very similar values between univariate and multivariate analyses (−3.85, CI 95% [−6.38; −1.35] and −4.11, CI 95% [−6.78; −1.45] for NOx for low occupational levels in the univariate and multivariate analyses, respectively).

**Figure 2:**
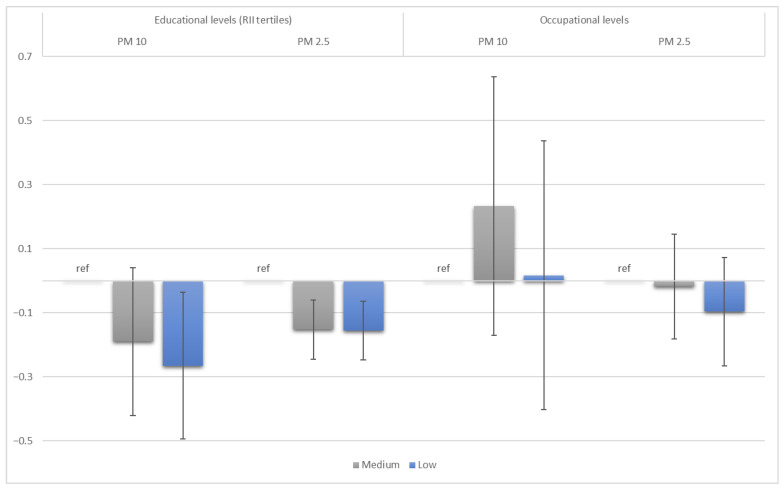
Beta coefficients (with 95% confidence intervals) of multivariate linear regression analysis with particulate matter as outcome and educational and occupational levels as exposures in Turin. The reference groups (ref) are the highly educated and the high-skilled occupational levels.

The analysis of the association between the SEP and mean levels of particulate matters in the Turin cohort revealed that it was very weak to null (Table 4). Indeed, in the univariable analysis, the beta coefficients for PM_10_ showed no association, and only the educational coefficient for PM_2.5_, although the coefficients were very small, were significant. In the multivariable analysis, the beta coefficient for PM_10_ was associated only among the low-educated (−0.27, 95% CI [−0.49; −0.04]), whereas all beta coefficients for PM_2.5_ exposure were significant, although with very small values (−0.15, CI 95% [−0.24; −0.06] and −0.16, CI 95% [−0.25; −0.06] for medium and low levels of education, respectively) (Figure 2).

## 4. Discussion

This study aimed to investigate the relationship between individual socioeconomic position, measured through educational or occupational level, and exposure to air pollution (NO_2_, NOx, PM_10_, and PM_2.5_) in the Turin and Varese EPIC cohorts. In the period 2008–2011, low-educated people and people with low-skilled jobs experienced slightly lower exposures to nitrogen molecule air pollution; no significant socioeconomic differences in particulate matter exposure were found in Turin.

As reported in a recent systematic review, the evidence on social inequalities in exposure to air pollution in Europe is mixed with an inconsistent pattern of association [12]. Indeed, published findings from different European cities return a motley picture in which the gradient can be direct, indirect, or not present at all. On the one hand, Paris [34], Vienna [35], Dortmund [36], Barcelona [37], Oslo [38], and other cities in the Netherlands [39] and England (including the city of London) [39] were characterised by a gradient according to which the more deprived areas were those with the highest air pollution. On the other hand, Rotterdam [39] and Bristol [39], along with Brussels [40], were not characterised by any gradient. In Rome, instead, an indirect gradient existed, according to which more affluent people experienced higher levels of air pollution compared to their low socioeconomic counterparts [41,42]. Finally, in Helsinki, Finland, the relationship between SEP and air pollution followed a U-shaped curve, with people in the highest and lowest socioeconomic positions exposed to higher air pollution levels and people in the middle with lower air pollution exposure [43]. From this collection of evidence, it emerges that there is not a common pattern [11]; instead, differences in the relationship between air pollution and deprivation across areas exist [44]. It can be argued that this relationship is rather shaped by the environmental, demographic, social, and geographic characteristics of the cities (i.e., where the more affluent sections of the population live) and by the urban policies on traffic and mobility. Indeed, whilst particulate matter comes from a number of possible sources [17] and exhibits a widespread prevalence, nitrogen molecules are associated with combustion sources and exhausts from vehicular traffic [17]. As a result, the combination of the social geography and the urban mobility strategies of a city may play a crucial role in defining the form and the direction of the relationship between SEP and exposure to air pollution.

**Figure A1:**
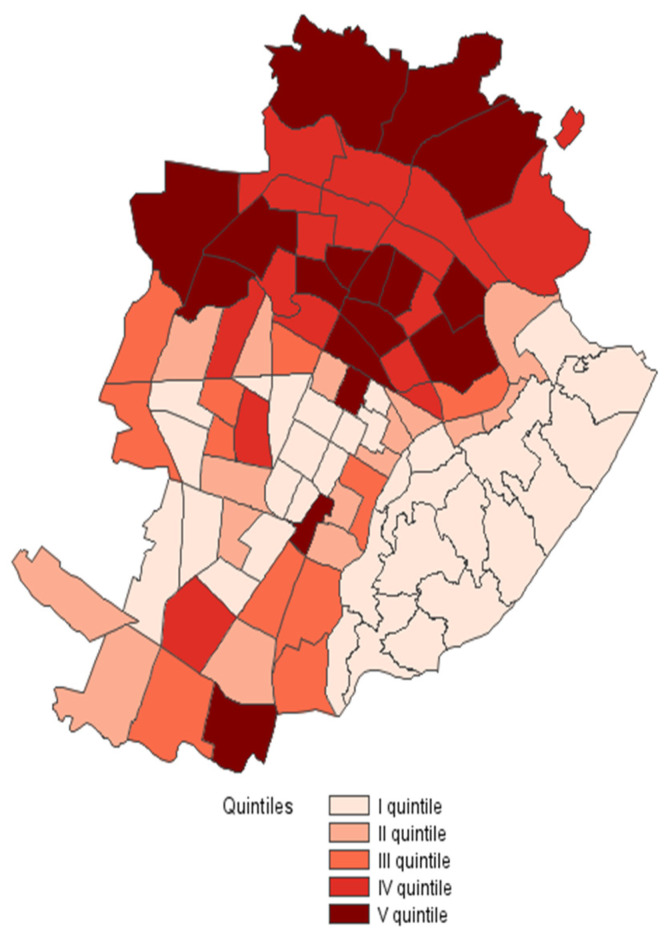
Distribution of statistical areas according to quintiles of the National Index of Deprivation, Turin [69].

This hypothesis is useful in explaining the results of our study. Indeed, both in Turin (see Figure A1 in Appendix A) and in the province of Varese, Italy’s sixth most densely populated province [45], with a polycentric structure, lacking a singular metropolis but featuring several sizable towns, the wealthier portions of the population (higher-educated and high-skilled employees) tend to live within the central districts—those with the highest level of traffic and therefore concentration of nitrogen molecules.

Although this spatial pattern could not be formally quantified within the scope of our study, previous research on the socioeconomic geography of Turin has shown that the majority of central neighbourhoods are inhabited by highly educated and affluent populations (see Figure A1 in Appendix A). This lends support to our qualitative interpretation that wealthier individuals may be more exposed to pollutants such as NO_2_ due to their concentration in traffic-heavy city centres. We acknowledge, however, that exceptions to this general pattern exist. In Turin, for example, some neighbourhoods immediately north of the historic centre are among the most socioeconomically deprived in the city, whereas affluent areas like the hillside district are located farther from the centre and are typically less exposed to vehicular pollution. Similarly, in the province of Varese, while the wealthier population segments are generally concentrated in central urban areas, several high-income municipalities lie outside the main urban cores.

Despite these exceptions, our results and interpretation are consistent with those described in the Turin children of the NINFEA cohort (an Italian web-based multi-purpose mother–child cohort based on questionnaires): even in this case, where SEP at childbirth was measured through the Equivalised Household Income Indicator, low–medium SEP children were less likely to be exposed to air pollution than the higher-SEP ones [46].

To lend support to the hypothesis that social geography and urban mobility interact in defining the relationship between SEP and exposure to air pollution, we collected information about major sources of air pollution and protective measures in the cities for which evidence on the relationship under study was available (Table 5). We collected data on city area, underground network length, underground network length per km square, cars per 1000 inhabitants in the city region, population, population density, and underground network length per 1000 city inhabitants. We grouped cities according to the type of association between deprivation and exposure to air pollution reported in the literature: direct proportionality, inverse proportionality, no relationship, and unique relationship. Although we did not find an evident correlation for any of the indicators, the underground kilometres relative to the city’s area and the number of cars per 1000 inhabitants in the region deserve a comment. The cities where the people in a lower SEP appeared to experience lower air pollution exposures (Turin and Rome) had a lower value for the first indicator and higher figures for the second compared to the cities where people in a lower SEP experienced higher air pollution levels. One possible interpretation is that a limited underground network coverage may lead to greater car dependence, which could contribute to widespread traffic congestion throughout the city, above all in central or affluent districts where higher-SEP individuals often reside. Conversely, in cities with more extensive public transport infrastructure, higher-SEP individuals may benefit more from protective urban planning, while lower-SEP populations may reside closer to pollution sources (e.g., high-traffic corridors). In approaching these data, it is important to bear in mind that the data reported are relative to the beginning of the year 2024; therefore, a perfect alignment with the studies’ times is not possible.

In the interpretation of the results of our study, in light of the current evidence, it is also important to mention that the relationship between SEP and exposure to air pollution could also change with respect to the use of different SEP indicators or the consideration of different city areas. For example, in Rome, the direction of the relationship has been demonstrated to reveal an opposite gradient (resulting in less affluent and less educated people being exposed to higher air pollution) if only the inner city centre is considered [42]. Another example of this variability due to the use of different SEP indicators (none of which, taken individually, consider all existing confounding variables [47]) was the situation described in Denmark, where the individual high level of education was associated with a higher level of air pollution, while neighbourhood socioeconomic indicators were not associated with air pollution [48]. Even in London, small-area markers of deprivation were associated with an increase in air pollution, but the association was reversed in central London and for SEP markers relating to education [49]. In addition, European data demonstrated that the power of the association reduced when passing from national to ecological to individual indicators of socioeconomic deprivation [12]. Therefore, our minimal gradient obtained using individual indicators was coherent.

Although the relationship between SEP and exposure to air pollution can show a pattern of reduced exposure among the socioeconomically disadvantaged strata of the population, other factors need to be taken into account when assessing the ultimate impact of air pollution on health. Indeed, besides the differences in exposure, the differences in susceptibility and vulnerability to air pollution play a crucial role in influencing health outcomes [12,50]. Demonstrating the importance of this second pathway, in Barcelona, the risk of dying due to environmental hazards in a very affluent neighbourhood was about 30% lower than in a very deprived one [37], and in the northwestern coastal region of England, the declaration of an Air Quality Management Area led to a larger decrease in hospitalisation rates in more compared to less deprived neighbourhoods [51]. Also, in Italy, increased exposure to particulate matter led to a more significant decrease in birth weight among children with less educated mothers than more educated ones [52], and one standard deviation increase in particulate matter exposure had a stronger effect in increasing hospitalisation among low-educated compared to more educated people [53]. Therefore, even though there was a minimal trend in favour of the more deprived for nitrogen molecules and similar exposure for particulate matter in our study areas, it is possible that these exposures contributed to health inequalities.

Therefore, considering this, policies aiming at reducing air pollution must look at health equity since the results could be counterintuitive. For example, in Paris, different scenarios of extension of the low-emission zone contributed to increased inequalities in preventable deaths and in new cases of three major chronic diseases [54]. Still, on the other hand, the positive example of the Athens reduction in air pollution led to greater mortality reduction among the more deprived, determining a reduction in health inequalities [55], as was also the case in the Scania province of Sweden [56]. Therefore, air pollution reduction must be carried out with equity in mind, considering that there is also no equity in emissions [57].

### Limitations and Strengths

This study suffered from a limitation because, despite being analysed as a cross-sectional study, air pollution level measurements were conducted in a period that was more than ten years later than the date of recruitment of the two cohorts: indeed, recruitment took place between 1993 and 1998, but measurements were conducted in the 2008–2010 and 2009–2011 periods. To consider them valid measures, two hypotheses should be true: that in those years, the reduction in air pollution occurred at the same rate in all parts of the city, and that in those years, recruited people remained at the same address. Both hypotheses could be considered valid: the first one, because neither in Turin nor in Varese has any specific policy to reduce air pollution in precise areas been implemented, and the second one, because recruited individuals were aged more than 35 years at enrolment, so they probably had already started a family, which may reduce the likelihood of further movement.

Another limitation of this study is its conclusions’ generalisation to the present: indeed, in the years between study recruitment and air pollution measurements, people with a higher SEP owned more powerful and more polluting cars, accounting for higher transport-related emissions [58]. This relationship is still being determined nowadays, with high income and high education now being positive determinants of owning low-emission cars [59,60], but also with data from London [61] and Paris [57] describing a situation in which the top emitters are people with the highest socioeconomic status. In addition, one must also consider the recent distribution of low-carbon heating systems, which favours the least deprived [62].

Moreover, as for most environmental studies, air pollution has been estimated at the residential addresses of the individuals in the cohort. This limitation was, in reality, a double one, since it considered only outdoor air pollution (but people spend the majority of their time indoors), and since people spent a lot of time in places other than home (for example, at work or at school). However, this limitation has been demonstrated to exerts little to no influence on the estimation of air pollution exposures in papers investigating this phenomenon [63,64].

Another limitation of this study was the way air pollutants were estimated: indeed, the developed LUR models were based only on 20 measurement sites for particulate matter and on 40 measurement sites for nitrogen molecules. Even if a strict rule for a minimum number of sites does not exist, a higher number of sites allows for a reduction in the risk of overfitting [23]. In addition, measurements were restricted in time (2008–2010 and 2009–2011), but previous sampling campaigns shared this limitation [23]. Furthermore, territory characteristics were collected in each city from current data, but the datasets might have been somewhat older [20,23].

Additionally, the cohort of Turin was composed of residents in high-population-density areas, the highest of which was the municipality of Turin itself, with a value of 6709.9 people per km^2^ in 2011 [65] (the first general census after our period of measurements). On the other hand, the province of Varese was characterised by very different territories, with a medium population density of 727.7 people per km^2^ in 2011 [66]. This could have been a problem since a higher population density could lead to higher air pollution levels [67]; however, this problem was addressed during analysis by stratifying results per cohort.

Another limitation of this study was that participants in the EPIC study volunteered, which may introduce biases if the selection process was associated with both SEP and air pollution. Indeed, people with a higher socioeconomic status are generally more likely to participate in such studies [68], creating a potential bias if our study cohorts were not geographically balanced: if a greater number of high- or low-educated people from specific areas participated, results could have been influenced. To limit possible distortions due to this, only relative measures were considered.

However, the voluntary enrolment allowed us to gather individual socio-demographic data, which is the main strength of this study. Indeed, two different SEP indicators were used, and the fact that the conclusions are rather similar gives strength to our findings.

Moreover, it also allowed us to estimate air pollution exposure at the address of each cohort member. Through this level of granularity, we achieved remarkable precision in our measurements, strengthening the reliability and accuracy of our data.

The final strength of this study lay in the high numerosity of its sample: the nearly 20,000 people considered enhanced the study’s statistical power, increasing the likelihood of detecting even minimal differences between groups.

## 5. Conclusions

In the Turin and Varese EPIC cohorts, a decade after enrolment, individuals with lower educational attainment and those with low-skilled occupations experienced slightly reduced exposure to nitrogen molecules compared to their counterparts in high-skilled jobs and with higher education levels. Conversely, there was a minimal, non-significant distinction between these two groups’ exposure to particulate matter in Turin. These findings, which reflect data from almost twenty years ago, still contribute to the progress in understanding the complex association between socioeconomic status and air pollution and should be analysed in conjunction with more recent studies, as well as studies on the effects of this exposure on populations’ health. Indeed, both exposure and susceptibility play an important role. Thus, even an exposure pattern in favour of the most deprived could conceal health consequences that favour the least deprived. Adopting this multifaceted approach will contribute to a more comprehensive understanding of the intricate relationship between air pollution and socioeconomic position, revealing any associated health implications.

## Figures and Tables

**Table 1:** Descriptive statistics (numbers, row percentages, and cumulative percentages) of all variables in the two cohorts.

		Varese		Turin	
	Category	Frequency	Percentage	Frequency	Percentage
**Educational levels (RII tertiles)**	High	3638	33.2	2689	31.3
	Medium	3118	28.4	2917	34.0
	Low	4212	38.4	2983	34.7
**Occupational levels**	High	496	6.0	507	6.7
	Medium	3516	42.5	4469	59.0
	Low	4263	51.5	2598	34.3
**Age**	35–44	2690	24.3	2384	27.2
	45–54	4440	40.1	3672	41.9
	55–64	3439	31.0	2689	30.7
	65+	517	4.7	11	0.1
**Sex**	Male	2369	21.4	4886	55.8
	Female	8717	78.6	3870	44.2
**Marital status**	Married	8853	86.8	6771	86.1
	Not Married	1349	13.2	1090	13.9
**Total**		11,086			8756

**Table 2:** NO_2_, NOx, PM_10_, and PM_2.5_ mean, standard deviation (Std.Dev.), and median in µg/m^3^ for the two cohorts.

		Turin	Varese	Total
**NOx**	Mean	99.55	85.38	91.63
	Std. Dev.	26.31	41.99	36.61
	Median	96.22	95.72	96.12
**NO_2_**	Mean	54.16	43.18	48.03
	Std. Dev.	11.66	17.35	16.06
	Median	55.20	47.74	51.98
**PM_10_**	Mean	46.51		
	Std. Dev.	4.17		
	Median	47.16		
**PM_2.5_**	Mean	30.14		
	Std. Dev.	1.67		
	Median	30.34		

**Table 3:** Descriptive statistics (mean and standard deviation, SD) of NO_2_ and NOx for every other variable of interest, *p*-values from *t*-tests or ANOVA tests, univariable beta coefficients, and 95% confidence intervals.

			NO_2_						NOx					
			Descriptives			Univariate Regression			Descriptives			Univariate Regression		
			Mean	SD	*p*-Value	Beta	CI 95%		Mean	SD	*p*-Value	Beta	CI 95%	
Varese	RII tertiles	High	43.96	17.57	<0.01 *	ref.			87.38	42.57	<0.01 *	ref.		
		Medium	43.45	17.08		−0.51	−1.34	0.32	86.00	41.42		−1.39	−3.39	0.62
		Low	42.29	17.29		**−1.67**	**−2.44**	**−0.90**	83.15	41.76		**−4.23**	**−6.09**	**−2.37**
	Occupational levels	High	46.68	16.60	<0.01 *	ref.			93.67	40.35	<0.01 *	ref.		
		Medium	42.26	17.48		**−4.42**	**−6.03**	**−2.81**	83.33	42.31		**−10.33**	**−14.23**	**−6.44**
		Low	43.52	16.92		**−3.15**	**−4.75**	**−1.56**	86.05	40.89		**−7.62**	**−11.48**	**−3.76**
	Age	35–44	40.36	17.44	<0.01 *	ref.			78.65	42.21	<0.01 *	ref.		
		45–54	43.30	17.20		2.95	2.12	3.77	85.61	41.53		6.96	4.96	8.96
		55–64	44.81	17.19		4.46	3.58	5.33	89.29	41.73		10.64	8.54	12.75
		65+	45.97	17.38		5.61	3.99	7.24	92.33	42.04		13.68	9.75	17.61
	Sex	Male	44.43	16.61	<0.01 **	ref.			88.20	40.23	<0.01 **	ref.		
		Female	42.84	17.53		−1.59	−2.37	−0.80	84.61	42.43		−3.60	−5.50	−1.69
	Marital status	Married	43.26	17.38	0.0186 **	ref.			85.56	42.05	0.015 **	ref.		
		Not married	44.45	17.19		1.19	0.19	2.18	88.53	41.71		2.97	0.56	5.38
Turin	RII tertiles	High	55.56	12.17	<0.01 *	ref.			101.80	27.26	<0.01 *	ref.		
		Medium	53.51	11.57		**−2.05**	**−2.66**	**−1.44**	98.23	25.94		**−3.57**	**−4.95**	**−2.19**
		Low	53.54	11.17		**−2.02**	**−2.62**	**−1.41**	98.82	25.76		**−2.98**	**−4.35**	**−1.61**
	Occupational levels	High	55.85	12.36	<0.01 *	ref.			102.77	28.80	<0.01 *	ref.		
		Medium	54.54	11.80		**−1.31**	**−2.39**	**−0.23**	99.93	26.29		**−2.84**	**−5.28**	**−0.39**
		Low	53.38	11.49		**−2.47**	**−3.59**	**−1.35**	98.92	26.75		**−3.85**	**−6.38**	**−1.32**
	Age	35–44	54.09	11.84	0.5271 *	ref.			99.59	27.06	0.4816 *	ref.		
		45–54	54.01	11.56		−0.08	−0.68	0.52	99.28	25.83		−0.31	−1.66	1.05
		55–64	54.42	11.61		0.32	−0.32	0.97	99.83	26.19		0.25	−1.20	1.70
		65+	56.02	17.14		1.93	−4.98	8.83	110.07	44.30		10.48	−5.11	26.06
	Sex	Male	54.17	11.73	0.9513 **	ref.			99.56	26.35	0.9527 **	ref.		
		Female	54.15	11.57		−0.02	−0.51	0.48	99.53	26.26		−0.03	−1.14	1.08
	Marital status	Married	53.81	11.68	0.0013 **	ref.			98.85	26.01	0.01 **	ref.		
		Not married	55.02	11.52		1.21	0.47	1.96	101.11	27.01		2.26	0.59	3.93

* ANOVA test; ** *t*-test. Bold indicates statistical significance in explanatory variables.

**Table 4:** Descriptive statistics (mean and standard deviation, Std.Dev.) of PM_2.5_ and PM_10_ for every other variable of interest, *p*-values from *t*-tests or ANOVA tests, univariable beta coefficients, and 95% confidence intervals.

			PM_2.5_						PM_10_					
			Descriptives			Univariate Regression			Descriptives			Univariate Regression		
			Mean	SD	*p*-Value	Beta	CI 95%		Mean	SD	*p*-Value	Beta	CI 95%	
Turin	RII tertiles	High	30.24	1.91	<0.01 *	ref.			46.66	4.54	0.1012 *	ref.		
		Medium	30.08	1.60		**−0.16**	**−0.25**	**−0.08**	46.45	4.07		−0.21	−0.43	0.01
		Low	30.11	1.49		**−0.13**	**−0.21**	**−0.04**	46.46	3.89		−0.20	−0.42	0.02
	Occupational levels	High	30.19	1.98	0.3846 *	ref.			46.30	4.64	0.1343 *	ref.		
		Medium	30.16	1.76		−0.02	−0.18	0.13	46.58	4.30		0.29	−0.10	0.67
		Low	30.11	1.48		−0.08	−0.24	0.08	46.42	3.84		0.12	−0.28	0.52
	Age	35–44	30.08	1.65	0.1752 *	ref.			46.38	4.24	0.0645 *	ref.		
		45–54	30.14	1.67		0.06	−0.02	0.15	46.47	4.15		0.08	−0.13	0.30
		55–64	30.18	1.68		0.10	0.01	0.19	46.67	4.12		0.29	0.06	0.52
		65+	30.38	1.42		0.29	−0.69	1.28	47.57	3.22		1.19	−1.28	3.66
	Sex	Male	30.12	1.68	0.1485 **	ref.			46.41	4.21	<0.01 **	ref.		
		Female	30.17	1.66		0.05	−0.02	0.12	46.64	4.10		0.23	0.06	0.41
	Marital status	Married	30.08	1.69	<0.01 **	ref.			46.37	4.21	<0.01 **	ref.		
		Not married	30.29	1.56		0.21	0.11	0.32	46.86	3.84		0.49	0.22	0.76

* ANOVA. ** *t*-test. Bold indicates statistical significance in explanatory variables.

**Table 5:** Major sources of air pollution and protective measures in European cities divided by the relationship between socioeconomic deprivation and air pollution.

Relationship	City	City Area (km^2^)	Underground Network (km)	Underground km/City Area	Cars per 1000 Inhabitants	Population	Population Density	Underground km per 1000 Inhabitants
Directly proportional	Paris	105.4	226.9	2.152751423	430	2,145,906	20,359.64	0.105736225
	Vienna	414.6	83.3	0.200916546	375	1,982,442	4781.58	0.042018884
	Dortmund	280.7	75.0	0.267189170	592	586,852	2090.60	0.127800536
	Barcelona	101.9	157.8	1.548577036	459	1,636,193	16,151.95	0.096443390
	Oslo	454.0	85.0	0.187224670	523	702,543	1547.45	0.120989036
	Amsterdam	219.3	45.0	0.205198358	434	921,402	4200.98	0.048838618
	London	1572.2	402.0	0.255700792		8,799,800	5597.30	0.045682856
No relationship	Rotterdam	324.1	102.3	0.315643320	437	639,587	2002.78	0.159946966
	Bristol	109.6	0.0	0.000000000		463,400	4228.10	0.000000000
	Brussels	178.7	55.7	0.311625825	402	2,708,766	15,154.78	0.020562869
Inversely proportional	Rome	1287.4	60.0	0.046607010	667	2,751,125	2137.03	0.021809260
	Turin	130.0	15.1	0.116144912	682	844,048	6945.18	0.017889978
U-shaped	Helsinki	213.8	43.0	0.201122544	539	664,028	3105.84	0.064756305

**Figure A2:**
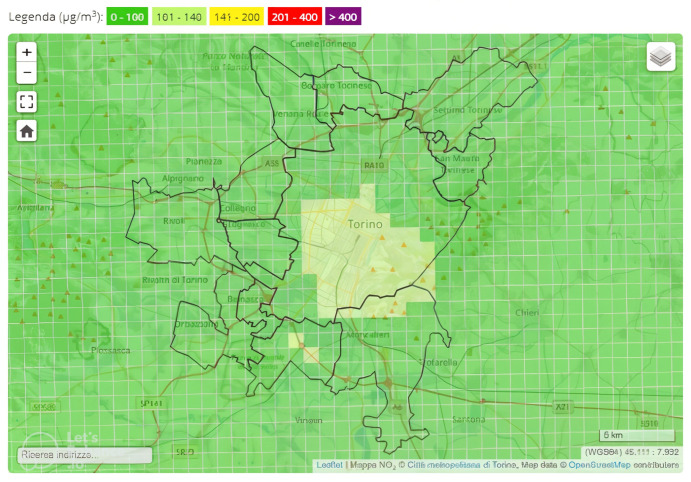
NO_2_ distribution in the Turin conurbation on a working day of December 2023 [70].

**Figure A3:**
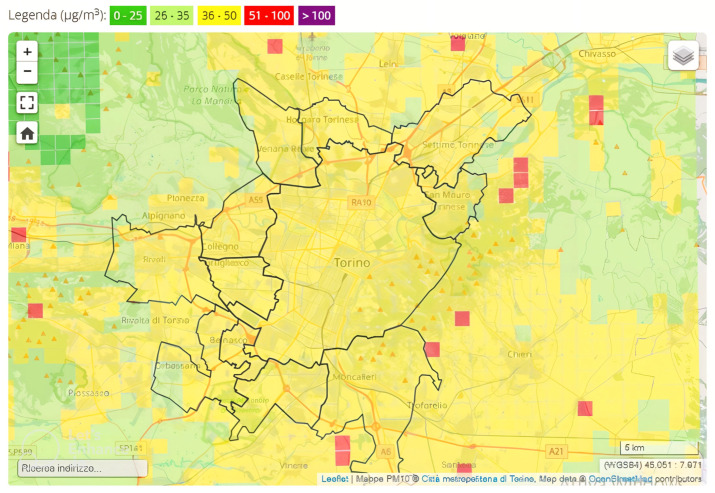
PM_10_ distribution in the Turin conurbation on a working day of December 2023 [70].

## Data Availability

The raw data cannot be made freely available because of restrictions imposed by the Ethical Committee that do not allow the open/public sharing of the data of individuals. However, aggregated data are available for other researchers upon request. Requests should be sent to: fulvio.ricceri@unito.it.

## Abbreviations

The following abbreviations are used in this manuscript:

EEA	European Environmental Agency
EPIC	European Prospective Investigation into Cancer and Nutrition
ESCAPE	European Study Cohorts for Air Pollution Effects
IARC	International Agency Against Cancer
ILO	International Labour Organization
ISCO	International Standard Classification of Occupations
NO_2_	Nitrogen dioxide
NOx	Nitrogen oxides
PM_10_	Particulate matter with an aerodynamic diameter of less than 10 μm
PM_2.5_	Particulate matter with an aerodynamic diameter of less than 2.5 μm
RII	Relative index of inequality
SEP	Socioeconomic position
WHO	World Health Organization

## Appendix A

**Table A1:** Cross-tabulation (numbers and row percentages) of educational level (RII tertiles) with the other variables of interest and *p*-values from chi-square test.

			Educational Levels (RII Tertiles)						
			High		Medium		Low		X^2^ *p*-Value
			No.	%	No.	%	No.	%	
Occupational Levels	Varese	High	352	71.8	90	18.4	48	9.8	<0.01
		Medium	2085	59.9	1035	29.7	363	10.4	
		Low	541	12.8	1166	27.7	2508	59.5	
	Turin	High	325	65.3	105	21.1	68	13.7	<0.01
		Medium	1947	44.2	1758	40.0	696	15.8	
		Low	133	5.3	751	29.6	1650	65.1	
	Total	High	677	68.5	195	19.7	116	11.7	<0.01
		Medium	4032	51.1	2793	35.4	1059	13.4	
		Low	674	10.0	1917	28.4	4158	61.6	
Age	Varese	35–44	1098	41.2	944	35.4	622	23.4	<0.01
		45–54	1286	29.3	1456	33.1	1655	37.6	
		55–64	1054	31.0	700	20.6	1642	48.4	
		65+	200	39.1	18	3.5	293	57.3	
	Turin	35–44	565	24.2	1063	45.5	709	30.3	<0.01
		45–54	1232	34.1	1109	30.7	1271	35.2	
		55–64	889	33.8	743	28.3	997	37.9	
		65+	3	27.3	2	18.2	6	54.6	
	Total	35–44	1663	33.3	2007	40.1	1331	26.6	<0.01
		45–54	2518	31.4	2565	32.0	2926	36.5	
		55–64	1943	32.3	1443	24.0	2639	43.8	
		65+	203	38.9	20	3.8	299	57.3	
Sex	Varese	Male	826	35.0	787	33.4	746	31.6	<0.01
		Female	2812	32.7	2331	27.1	3466	40.3	
	Turin	Male	1314	27.4	1855	38.7	1630	34.0	<0.01
		Female	1375	36.3	1062	28.0	1353	35.7	
	Total	Male	2140	29.9	2642	36.9	2376	33.2	<0.01
		Female	4187	33.8	3393	27.4	4819	38.9	
Marital status	Varese	Married	2811	32.0	2545	29.0	3422	39.0	<0.01
		Not married	550	41.5	279	21.0	497	37.5	
	Turin	Married	2117	31.8	2256	33.9	2278	34.3	<0.01
		Not married	391	36.4	385	35.8	299	27.8	
	Total	Married	4928	31.9	4801	31.1	5700	36.9	<0.01
		Not married	941	39.2	664	27.7	796	33.2	

**Table A2:** Cross-tabulation (numbers and row percentages) of occupational levels with the other variables of interest and *p*-values from chi-square test.

			Occupational Levels						
			High		Medium		Low		X^2^ *p*-Value
			No.	%	No.	%	No.	%	
Age	Varese	35–44	117	5.5	1223	56.9	808	37.6	<0.01
		45–54	160	5.3	1273	42.3	1576	52.4	
		55–64	201	7.3	883	32.2	1656	60.4	
		65+	18	4.8	137	36.2	223	59.0	
	Turin	35–44	151	7.0	1362	62.8	655	30.2	<0.01
		45–54	198	6.4	1935	62.2	979	31.5	
		55–64	158	6.9	1167	51.1	958	42.0	
		65+	0	0.0	5	45.5	6	54.6	
	Total	35–44	268	6.2	2585	59.9	1463	33.9	<0.01
		45–54	358	5.9	3208	52.4	2555	41.7	
		55–64	359	7.2	2050	40.8	2614	52.0	
		65+	18	4.6	142	36.5	229	58.9	
Sex	Varese	Male	282	12.0	811	34.4	1264	53.6	<0.01
		Female	214	3.6	2705	45.7	2999	50.7	
	Turin	Male	378	7.8	2710	55.8	1769	36.4	<0.01
		Female	129	4.8	1759	64.7	829	30.5	
	Total	Male	660	9.2	3521	48.8	3033	42.0	<0.01
		Female	343	4.0	4464	51.7	3828	44.3	
Marital status	Varese	Married	384	6.1	2676	42.7	3202	51.1	0.01
		Not married	46	4.1	516	45.6	570	50.4	
	Turin	Married	375	6.5	3428	59.5	1955	34.0	<0.01
		Not married	84	8.5	644	64.8	266	26.8	
	Total	Married	759	6.3	6104	50.8	5157	42.9	<0.01
		Not married	130	6.1	1160	54.6	836	39.3	

**Table A3:** Multivariable beta coefficients and 95% confidence intervals for NO_2_ and NOx.

			NO_2_						NOx					
			Multivariate Regression						Multivariate Regression					
			Beta	CI 95%		Beta	CI 95%		Beta	CI 95%		Beta	CI 95%	
Varese	RII tertiles	High	ref.						ref.					
		Medium	−0.59	−1.47	0.28				−1.56	−3.67	0.54			
		Low	−2.34	−3.14	−1.53				−5.85	−7.80	−3.89			
	Occupational levels	High				ref.						ref.		
		Medium				−3.32	−5.06	−1.57				−7.82	−12.04	−3.60
		Low				−3.14	−4.87	−1.42				−7.72	−11.89	−3.55
	Age	35–44	ref.			ref.			ref.			ref.		
		45–54	3.01	2.13	3.88	2.92	1.91	3.94	7.13	5.01	9.25	6.95	4.49	9.41
		55–64	4.01	3.05	4.96	4.03	2.97	5.08	9.65	7.35	11.96	9.76	7.20	12.32
		65+	5.74	4.04	7.45	5.84	3.91	7.77	14.02	9.88	18.15	14.35	9.68	19.02
	Sex	Male	ref.			ref.			ref.			ref.		
		Female	−3.13	−4.10	−2.15	−3.19	−4.22	−2.17	−7.21	−9.58	−4.84	−7.38	−9.85	−4.90
	Marital status	Married	ref.			ref.			ref.			ref.		
		Not married	0.89	−0.13	1.91	1.09	−0.01	2.19	2.20	−0.27	4.67	2.61	−0.06	5.28
Turin	RII tertiles	High	ref.						ref.					
		Medium	−2.19	−2.84	−1.55				−3.94	−5.39	−2.49			
		Low	−2.16	−2.80	−1.52				−3.40	−4.84	−1.96			
	Occupational levels	High				ref.						ref.		
		Medium				−1.30	−2.43	−0.16				−2.86	−5.42	−0.29
		Low				−2.54	−3.72	−1.36				−4.11	−6.78	−1.45
	Age	35–44	ref.			ref.			ref.			ref.		
		45–54	−0.02	−0.66	0.63	0.09	−0.59	0.78	−0.20	−1.65	1.25	0.39	−1.15	1.93
		55–64	0.29	−0.40	0.99	0.38	−0.36	1.12	0.11	−1.45	1.67	0.18	−1.49	1.85
		65+	2.98	−5.65	11.61	2.65	−6.07	11.37	10.28	−9.14	2.97	9.59	−10.07	29.25
	Sex	Male	ref.			ref.			ref.			ref.		
		Female	−0.20	−0.72	0.33	0.06	−0.53	0.65	−0.51	−1.70	0.68	0.32	−1.00	1.65
	Marital status	Married	ref.			ref.			ref.			ref.		
		Not married	1.07	0.32	1.83	1.25	0.45	2.06	2.00	0.29	3.70	2.17	0.36	3.99

**Table A4:** Multivariable beta coefficients and 95% confidence intervals for PM_10_ and PM_2.5_.

			PM_2.5_						PM_10_					
			Multivariate Regression						Multivariate Regression					
Turin	RII tertiles	High	ref.						ref.					
		Medium	−0.15	−0.24	−0.06				−0.19	−0.42	0.04			
		Low	−0.16	−0.25	−0.06				−0.27	−0.49	−0.04			
	Occupational levels	High				ref.						ref.		
		Medium				−0.02	−0.18	0.14				0.23	−0.17	0.64
		Low				−0.10	−0.27	0.07				0.02	−0.40	0.44
	Age	35–44	ref.			ref.			ref.			ref.		
		45–54	0.09	−0.01	0.18	0.11	0.02	0.21	0.12	−0.11	0.36	0.07	−0.17	0.31
		55–64	0.10	0.00	0.20	0.12	0.01	0.22	0.31	0.06	0.56	0.26	0.00	0.52
		65+	0.49	−0.75	1.73	0.45	−0.80	1.70	1.27	−1.82	4.36	1.15	−1.94	4.24
	Sex	Male	ref.			ref.			ref.			ref.		
		Female	0.05	−0.03	0.12	0.07	−0.01	0.16	0.25	0.06	0.44	0.26	0.05	0.47
	Marital status	Married	ref.			ref.			ref.			ref.		
		Not married	0.20	0.09	0.31	0.22	0.11	0.34	0.43	0.16	0.70	0.52	0.24	0.81

## References

[1] WHO Air Pollution—WHO. https://www.who.int/health-topics/air-pollution#tab=tab_2.

[2] Laurent É. (2022). Air (Ine) Quality in the European Union. Curr. Environ. Health Rep..

[3] WHO What Are the WHO Air Quality Guidelines?. https://www.who.int/news-room/feature-stories/detail/what-are-the-who-air-quality-guidelines.

[4] European Union (2008). Directive 2008/50/EC of the European Parliament and of the Council of 21 May 2008 on Ambient Air Quality and Cleaner Air for Europe.

[5] WHO (2022). European Commission Proposal for a Directive of the European Parliament and of the Council on Ambient Air Quality and Cleaner Air for Europe.

[6] EEA Annual Reports—EEA. https://www.eea.europa.eu/about-us/documents/annual-reports.

[7] EEA Europe’s Air Quality Status 2023. https://www.eea.europa.eu/publications/europes-air-quality-status-2023.

[8] EEA Harm to Human Health from Air Pollution in Europe: Burden of Disease 2023. https://www.eea.europa.eu/publications/harm-to-human-health-from-air-pollution/.

[9] OECD Health Inequalities—OECD. https://www.oecd.org/health/inequalities-in-health.htm.

[10] WHO Health Inequities and Their Causes. https://www.who.int/news-room/facts-in-pictures/detail/health-inequities-and-their-causes.

[11] Hajat A., Hsia C., O’Neill M.S. (2015). Socioeconomic Disparities and Air Pollution Exposure: A Global Review. Curr. Environ. Health Rep..

[12] Fairburn J., Schüle S.A., Dreger S., Karla Hilz L., Bolte G. (2019). Social Inequalities in Exposure to Ambient Air Pollution: A Systematic Review in the WHO European Region. Int. J. Environ. Res. Public Health.

[13] Vineis P., Hoek G., Krzyzanowski M., Vigna-Taglianti F., Veglia F., Airoldi L., Autrup H., Dunning A., Garte S., Hainaut P. (2006). Air Pollution and Risk of Lung Cancer in a Prospective Study in Europe. Int. J. Cancer.

[14] Bingham S., Riboli E. (2004). Diet and Cancer—The European Prospective Investigation into Cancer and Nutrition. Nat. Rev. Cancer.

[15] IARC EPIC Study. International Agency for Research on Cancer: Lyon, France. https://epic.iarc.fr/centres/italy/.

[16] Raaschou-Nielsen O., Andersen Z.J., Beelen R., Samoli E., Stafoggia M., Weinmayr G., Hoffmann B., Fischer P., Nieuwenhuijsen M.J., Brunekreef B. (2013). Air Pollution and Lung Cancer Incidence in 17 European Cohorts: Prospective Analyses from the European Study of Cohorts for Air Pollution Effects (ESCAPE). Lancet Oncol..

[17] IARC (2015). Outdoor Air Pollution. IARC Monographs on the Evaluation of Carcinogenic Risks to Humans.

[18] Beelen R., Raaschou-Nielsen O., Stafoggia M., Andersen Z.J., Weinmayr G., Hoffmann B., Wolf K., Samoli E., Fischer P., Nieuwenhuijsen M. (2014). Effects of Long-Term Exposure to Air Pollution on Natural-Cause Mortality: An Analysis of 22 European Cohorts within the Multicentre ESCAPE Project. Lancet.

[19] Cyrys J., Eeftens M., Heinrich J., Ampe C., Armengaud A., Beelen R., Bellander T., Beregszaszi T., Birk M., Cesaroni G. (2012). Variation of NO_2_ and NOx Concentrations between and within 36 European Study Areas: Results from the ESCAPE Study. Atmos. Environ..

[20] Eeftens M., Tsai M.-Y., Ampe C., Anwander B., Beelen R., Bellander T., Cesaroni G., Cirach M., Cyrys J., De Hoogh K. (2012). Spatial Variation of PM2.5, PM10, PM2.5 Absorbance and PMcoarse Concentrations between and within 20 European Study Areas and the Relationship with NO_2_—Results of the ESCAPE Project. Atmos. Environ..

[21] Hoek G., Meliefste K., Cyrys J., Lewné M., Bellander T., Brauer M., Fischer P., Gehring U., Heinrich J., Van Vliet P. (2002). Spatial Variability of Fine Particle Concentrations in Three European Areas. Atmos. Environ..

[22] Hoek G., Beelen R., De Hoogh K., Vienneau D., Gulliver J., Fischer P., Briggs D. (2008). A Review of Land-Use Regression Models to Assess Spatial Variation of Outdoor Air Pollution. Atmos. Environ..

[23] Eeftens M., Beelen R., De Hoogh K., Bellander T., Cesaroni G., Cirach M., Declercq C., Dėdelė A., Dons E., De Nazelle A. (2012). Development of Land Use Regression Models for PM_2.5_, PM_2.5_ Absorbance, PM_10_ and PM_coarse_ in 20 European Study Areas; Results of the ESCAPE Project. Environ. Sci. Technol..

[24] Beelen R., Hoek G., Vienneau D., Eeftens M., Dimakopoulou K., Pedeli X., Tsai M.-Y., Künzli N., Schikowski T., Marcon A. (2013). Development of NO_2_ and NOx Land Use Regression Models for Estimating Air Pollution Exposure in 36 Study Areas in Europe—The ESCAPE Project. Atmos. Environ..

[25] Galobardes B. (2006). Indicators of Socioeconomic Position (Part 1). J. Epidemiol. Community Health.

[26] Mackenbach J.P., Kunst A.E. (1997). Measuring the Magnitude of Socio-Economic Inequalities in Health: An Overview of Available Measures Illustrated with Two Examples from Europe. Soc. Sci. Med..

[27] Sacerdote C., Ricceri F., Rolandsson O., Baldi I., Chirlaque M.-D., Feskens E., Bendinelli B., Ardanaz E., Arriola L., Balkau B. (2012). Lower Educational Level Is a Predictor of Incident Type 2 Diabetes in European Countries: The EPIC-InterAct Study. Int. J. Epidemiol..

[28] Fujishiro K., Xu J., Gong F. (2010). What Does “Occupation” Represent as an Indicator of Socioeconomic Status?: Exploring Occupational Prestige and Health. Soc. Sci. Med..

[29] ISCO (2008). International Standard Classification of Occupations.

[30] d’Errico A., Ricceri F., Stringhini S., Carmeli C., Kivimaki M., Bartley M., McCrory C., Bochud M., Vollenweider P., Tumino R. (2017). Socioeconomic Indicators in Epidemiologic Research: A Practical Example from the LIFEPATH Study. PLoS ONE.

[31] George D., Mallery P. (2010). SPSS for Windows Step by Step: A Simple Guide and Reference, 17.0 Update.

[32] Hair J.F., Black W.C., Babin B.J., Anderson R.E., Hair J.F. (2010). Multivariate Data Analysis.

[33] Byrne B.M. (2010). Structural Equation Modeling with AMOS: Basic Concepts, Applications, and Programming.

[34] Kihal-Talantikite W., Legendre P., Le Nouveau P., Deguen S. (2018). Premature Adult Death and Equity Impact of a Reduction of NO_2_, PM10, and PM2.5 Levels in Paris—A Health Impact Assessment Study Conducted at the Census Block Level. Int. J. Environ. Res. Public Health.

[35] Khomenko S., Nieuwenhuijsen M., Ambròs A., Wegener S., Mueller N. (2020). Is a Liveable City a Healthy City? Health Impacts of Urban and Transport Planning in Vienna, Austria. Environ. Res..

[36] Flacke J., Schüle S., Köckler H., Bolte G. (2016). Mapping Environmental Inequalities Relevant for Health for Informing Urban Planning Interventions—A Case Study in the City of Dortmund, Germany. Int. J. Environ. Res. Public Health.

[37] Saez M., López-Casasnovas G. (2019). Assessing the Effects on Health Inequalities of Differential Exposure and Differential Susceptibility of Air Pollution and Environmental Noise in Barcelona, 2007–2014. Int. J. Environ. Res. Public Health.

[38] Venter Z.S., Figari H., Krange O., Gundersen V. (2023). Environmental Justice in a Very Green City: Spatial Inequality in Exposure to Urban Nature, Air Pollution and Heat in Oslo, Norway. Sci. Total Environ..

[39] Fecht D., Fischer P., Fortunato L., Hoek G., De Hoogh K., Marra M., Kruize H., Vienneau D., Beelen R., Hansell A. (2015). Associations Between Air Pollution and Socioeconomic Characteristics, Ethnicity and Age Profile of Neighbourhoods in England and the Netherlands. Environ. Pollut..

[40] Da Schio N., Boussauw K., Sansen J. (2019). Accessibility versus Air Pollution: A Geography of Externalities in the Brussels Agglomeration. Cities.

[41] Badaloni C., De Sario M., Caranci N., De’ Donato F., Bolignano A., Davoli M., Leccese L., Michelozzi P., Leone M. (2023). A Spatial Indicator of Environmental and Climatic Vulnerability in Rome. Environ. Int..

[42] Cesaroni G., Badaloni C., Romano V., Donato E., Perucci C.A., Forastiere F. (2010). Socioeconomic Position and Health Status of People Who Live near Busy Roads: The Rome Longitudinal Study (RoLS). Environ. Health.

[43] Gosztonyi Á., Demmler J.C., Juhola S., Ala-Mantila S. (2023). Ambient Air Pollution-Related Environmental Inequality and Environmental Dissimilarity in Helsinki Metropolitan Area, Finland. Ecol. Econ..

[44] Bailey N., Dong G., Minton J., Pryce G. (2018). Reconsidering the Relationship between Air Pollution and Deprivation. Int. J. Environ. Res. Public Health.

[45] AdminSTAT Mappe Tematiche, Curiosità, Confronti e Classifiche per i Comuni, Le Province e Le Regioni Sulla Base Di 20 Indicatori Socio-Demografici. https://ugeo.urbistat.com/adminstat/it/it/classifiche/dati-sintesi/regioni/italia/380/1.

[46] Moccia C., Pizzi C., Moirano G., Popovic M., Zugna D., d’Errico A., Isaevska E., Fossati S., Nieuwenhuijsen M.J., Fariselli P. (2023). Modelling Socioeconomic Position as a Driver of the Exposome in the First 18 Months of Life of the NINFEA Birth Cohort Children. Environ. Int..

[47] Sørensen M., Hvidtfeldt U.A., Poulsen A.H., Thygesen L.C., Frohn L.M., Ketzel M., Christensen J.H., Brandt J., Khan J., Raaschou-Nielsen O. (2022). The Effect of Adjustment to Register-Based and Questionnaire-Based Covariates on the Association between Air Pollution and Cardiometabolic Disease. Environ. Res..

[48] Raaschou-Nielsen O., Taj T., Poulsen A.H., Hvidtfeldt U.A., Ketzel M., Christensen J.H., Brandt J., Frohn L.M., Geels C., Valencia V.H. (2022). Air Pollution at the Residence of Danish Adults, by Socio-Demographic Characteristics, Morbidity, and Address Level Characteristics. Environ. Res..

[49] Goodman A., Wilkinson P., Stafford M., Tonne C. (2011). Characterising Socio-Economic Inequalities in Exposure to Air Pollution: A Comparison of Socio-Economic Markers and Scales of Measurement. Health Place.

[50] Forastiere F., Stafoggia M., Tasco C., Picciotto S., Agabiti N., Cesaroni G., Perucci C.A. (2007). Socioeconomic Status, Particulate Air Pollution, and Daily Mortality: Differential Exposure or Differential Susceptibility. Am. J. Ind. Med..

[51] Rose T.C., Daras K., Cloke J., Rodgers S., Farrell P., Ahmed S., Barr B. (2021). Impact of Local Air Quality Management Policies on Emergency Hospitalisations for Respiratory Conditions in the North West Coast Region of England: A Longitudinal Controlled Ecological Study. Int. J. Equity Health.

[52] Palma A., Petrunyk I., Vuri D. (2022). Prenatal Air Pollution Exposure and Neonatal Health. Health Econ..

[53] Giaccherini M., Kopinska J., Palma A. (2021). When Particulate Matter Strikes Cities: Social Disparities and Health Costs of Air Pollution. J. Health Econ..

[54] Host S., Honoré C., Joly F., Saunal A., Le Tertre A., Medina S. (2020). Implementation of Various Hypothetical Low Emission Zone Scenarios in Greater Paris: Assessment of Fine-Scale Reduction in Exposure and Expected Health Benefits. Environ. Res..

[55] Bailey J., Gerasopoulos E., Rojas-Rueda D., Benmarhnia T. (2019). Potential Health and Equity Co-Benefits Related to the Mitigation Policies Reducing Air Pollution from Residential Wood Burning in Athens, Greece. J. Environ. Sci. Health Part A.

[56] Flanagan E., Stroh E., Oudin A., Malmqvist E. (2019). Connecting Air Pollution Exposure to Socioeconomic Status: A Cross-Sectional Study on Environmental Injustice among Pregnant Women in Scania, Sweden. Int. J. Environ. Res. Public Health.

[57] Leroutier M., Quirion P. (2022). Air Pollution and CO_2_ from Daily Mobility: Who Emits and Why? Evidence from Paris. Energy Econ..

[58] Büchs M., Schnepf S.V. (2013). Who Emits Most? Associations between Socio-Economic Factors and UK Households’ Home Energy, Transport, Indirect and Total CO_2_ Emissions. Ecol. Econ..

[59] Qorbani D., Korzilius H.P.L.M., Fleten S.-E. (2024). Ownership of Battery Electric Vehicles Is Uneven in Norwegian Households. Commun. Earth Environ..

[60] Mandys F. (2021). Electric Vehicles and Consumer Choices. Renew. Sustain. Energy Rev..

[61] Possible (2023). Inspiring Climate Action. Tractor Attack: Fairness in Pricing Traffic Pollution and Rising SUV Emissions in Kensington & Chelsea and Beyond.

[62] Burlison A., Davillas A., Giulietti M. (2023). We’re on the Road to Net Zero? Socioeconomic Inequality in Low-Carbon Technology Adoption.

[63] Setton E., Marshall J.D., Brauer M., Lundquist K.R., Hystad P., Keller P., Cloutier-Fisher D. (2011). The Impact of Daily Mobility on Exposure to Traffic-Related Air Pollution and Health Effect Estimates. J. Expo. Sci. Environ. Epidemiol..

[64] Ragettli M.S., Phuleria H.C., Tsai M.-Y., Schindler C., De Nazelle A., Ducret-Stich R.E., Ineichen A., Perez L., Braun-Fahrländer C., Probst-Hensch N. (2015). The Relevance of Commuter and Work/School Exposure in an Epidemiological Study on Traffic-Related Air Pollution. J. Expo. Sci. Environ. Epidemiol..

[65] ISTAT POPOLAZIONE|Dinamica Demografica e Territorio—Torino. https://ottomilacensus.istat.it/sottotema/001/001272/1/.

[66] ISTAT POPOLAZIONE|Dinamica Demografica e Territorio—Provincia Di Varese. https://ottomilacensus.istat.it/provincia/012/.

[67] Borck R., Schrauth P. (2021). Population Density and Urban Air Quality. Reg. Sci. Urban Econ..

[68] Niebuur J., Van Lente L., Liefbroer A.C., Steverink N., Smidt N. (2018). Determinants of Participation in Voluntary Work: A Systematic Review and Meta-Analysis of Longitudinal Cohort Studies. BMC Public Health.

[69] Rosano A., Pacelli B., Zengarini N., Costa G., Cislaghi C., Caranci N. (2020). Aggiornamento e revisione dell’ indice di deprivazione italiano 2011 a livello di sezione di censimento. EP.

[70] Città Metropolitana di Torino QUALITÀ DELL’ARIA INDICE PREVISIONALE DI QUALITÀ DELL’ARIA (IPQA). http://www.cittametropolitana.torino.it/cms/ambiente/qualita-aria/dati-qualita-aria/ipqa.

